# Support actions undertaken for the woman by companions in public maternity hospitals^1^

**DOI:** 10.1590/1518-8345.2251.2994

**Published:** 2018-06-21

**Authors:** Carolina Frescura Junges, Odaléa Maria Brüggemann, Roxana Knobel, Roberta Costa

**Affiliations:** 2 PhD, RN, Hospital Universitário Polydoro Ernani de São Thiago, Universidade Federal de Santa Catarina, Florianópolis, SC, Brazil.; 3 PhD, Associate Professor, Departamento de Enfermagem, Universidade Federal de Santa Catarina, Florianópolis, SC, Brazil.; 4 PhD, Adjunct Professor, Departamento de Ginecologia e Obstetrícia, Universidade Federal de Santa Catarina, Florianópolis, SC, Brazil.; 5 PhD, Adjunct Professor, Departamento de Enfermagem, Universidade Federal de Santa Catarina, Florianópolis, SC, Brazil.

**Keywords:** Humanizing Delivery, Social Support, Obstetric Nursing, Labor, Obstetric, Parturition, Women’s Health

## Abstract

**Objective:**

to identify the support actions undertaken for the woman during labor, birth, cesarean section and the postpartum period.

**Method:**

a transversal study, undertaken in three public maternity hospitals, with a sample of 1,147 companions. The data were collected through interviews and were analyzed using descriptive statistics. The support actions were classified in four dimensions: emotional, physical, informational and relating to intermediation.

**Results:**

the majority of interviewees were the partner/father of the baby (76.7%). In labor, birth and the postpartum period, the actions of emotional support - such as calming, encouraging and praising, were performed by more than 80.0% of the companions; informational support, by approximately 70.0%; and intermediation by fewer than 65.0% of them. In childbirth, the emphasis on physical support was observed in assisting with walking (84.4%), and in changing position (90.4%).

**Conclusion:**

the companions participate actively in the birth process, performing actions of support in the four dimensions. Emotional support is the most frequent, followed by physical and informational support, mainly during labor and birth. The results contribute to valuing the companion from the woman’s social network in the birth scenario and to the recognition of his/her role as a provider of support.

## Introduction

In Brazil, the transition of the birth scenario from the home to the hospital took place at the beginning of the 20th century. This process was a determinant for consolidating the technocratic view of birth, with the physician as the central figure - besides the broad use of procedures and interventions whose efficiency was not always proven, and not even always beneficial^1^ ^-^ ^2^. In this scenario, the presence of the family and of people from the parturient woman’s social network became unwanted, as it interfered negatively with the hospitals’ policies and routines. As a result, the family’s withdrawal, and the elimination of the emotional support for the woman during labor and birth, was inevitable^2^.

In the 1980s, women’s discontentment with the medicalization of birth, apart from other grievances, was one of the axes of debates for female protagonism. The women’s movement in Brazil, also conducted by the feminist current, achieved visibility and obtained many victories in the health area. Indeed, after the creation of the National Program for Integrated Healthcare for Women (*Programa de Assistência Integral à Saúde da Mulher*), an ideological strengthening for the humanization of labor and childbirth could be observed^3^ ^-^ ^4^. Agreeing with this movement, the recommendation of the World Health Organization (WHO) emphasizes the companion’s presence as one of the good practices in obstetrics, that is, a practice which minimizes the undertaking of interventions which are not proven to be beneficial^5^.

In Brazil, in 2005, upon the approval of Federal Law N. 11,108, women gained the legal right to have a companion of their choice during labor, birth and the immediate postpartum period^6^. This legal support aims to protect women’s rights, facilitating the companion’s remaining present during obstetric inpatient treatment. However, studies have identified that ignorance and failure to comply with this Law continue to be considerable^6^ ^-^ ^7^.

The Born in Brazil Survey (*Pesquisa Nascer no Brasil*), which interviewed 23,940 puerpural women, analyzed relevant aspects regarding the implementation of the right to a companion in the maternity hospitals. The total absence of the companion during obstetric inpatient treatment was cited by 24.5% of women; 18.8% had a companion continuously, and 56.7% had a companion present only at certain points of the inpatient treatment. The factors associated with the implementation of the right to a companion in the maternity hospital studied were: an appropriate environment and clear institutional rules regarding women’s rights^8^.

The continuous support provided by the companion is considered to be beneficial for the woman and the newborn, as it contributes to a reduction in the number of cesarean sections, in the duration of labor and in the number of interventions during labor and birth - and increases the women’s level of satisfaction with the experience^9^. Such evidence, together with other studies, also indicates the importance of embracing the man in the birth scenario, contributing to the support for the woman, the transition to fatherhood and the formation of an early bond with the newborn^10^ ^-^ ^11^.

The support actions undertaken by the birth companion may be classified in four dimensions: emotional, when the provider of support makes himself present continuously, and encourages, calms and praises the woman; physical, when he assists in the birthing pool, and changing position, in reducing pain and in massaging; informational, when he explains things to and informs the pregnant woman about what is happening; and, finally, intermediation - when he interprets and negotiates the woman’s wishes with the health professionals^9^. The actions of emotional and physical support are carried out most and, consequently, are remembered by the women and their birth companions^6^ ^,^ ^10^ ^,^ ^12^ ^-^ ^13^.

Most studies focus on the woman’s view of the benefits provided by the companion^12^ ^,^ ^14^ ^-^ ^16^, mainly based on studies with qualitative approaches. However, few works have provided companions with the opportunity to report which support actions they felt comfortable undertaking, or for which they received guidance on how to provide the support actions to the woman^6^ ^,^ ^13^. In the international scenario, most studies have not described the dimensions of the support actions undertaken by the companion, whether the companion was a family member, a doula, a midwife or nurse^9^. The present study, therefore, contributes to the construction of knowledge regarding this topic at a national and international level. 

In the Brazilian scenario, the maternity hospitals which comply with the Law allow the presence of the companion - this generally being a member of the woman’s family or social network^8^. In many cases, however, there is an understanding that the companion is a mere spectator. The support actions undertaken by the companion must be known in order to identify and value their real participation during the time they spend in the maternity hospital. Besides this, in strengthening the companion as a provider of support for the woman, a fresh look could be directed on this practice so that the health professionals may allow the companion to exercise their role.

In this way, this work’s objective was to identify the support actions undertaken by the companion during labor, birth, cesarean section and the postpartum period in public maternity hospitals in Grande Florianópolis, Santa Catarina, Brazil.

## Method

A transversal study which forms part of the macroproject titled “The participation of the companion of the woman’s choice in the prenatal care, labor and childbirth in the public and supplementary health systems”. 

The data were collected in the three public maternity hospitals in Grande Florianópolis, in the Brazilian state of Santa Catarina (SC), which attend women only through the Unified Health System (SUS). The study locales were termed Maternity Hospitals A, B and C. The three institutions are maternity hospitals with midwifery schools and host placements for undergraduate students in Nursing and Medicine; they also have a Medical Residency in Gynecology and Obstetrics and have agreements with the Stork Network (*Rede Cegonha*). Besides this, they provide written guidance for companions and have a bathroom with a shower, a birthing ball, and rocking stool (known as the ‘rocking horse’ because of its similarity to the child’s toy) for the parturient women. Maternity Hospital A has allowed the presence of the companion of the woman’s choice since 2000. Maternity Hospital B has allowed the presence of the companion of the woman’s choice since 2002, is recognized as a Child-Friendly Hospital (*Hospital Amigo da Criança*), is a center of excellence in the state for Women’s Health, and received the National Dr. Pinetti Prize for being a Woman-Friendly Hospital (*Prêmio Nacional Dr. Pinotti de Hospital Amigo da Mulher*) in 2013. Maternity Hospital C has allowed the presence of the companion of the woman’s choice since 1995, is a Child-Friendly Hospital, received the Galba de Araújo Prize (*Prêmio Galba de Araújo*) in 2000, is a national center of excellence in Humanized Care for the Low-Weight Newborn: Kangaroo Care and has a Multi-professional Residency with emphasis on Care for the Health of the Woman and Child.

The research subjects were the people who stayed with the women during labor and birth or the cesarean section. The inclusion criteria were as follows: to have remained with the woman in the maternity hospital during labor and birth or the cesarean section, regardless of the duration of each period. The exclusion criteria were: to have been the companion of a woman who underwent emergency or elective cesarean section, as labor did not take place and the companion did not have the opportunity to carry out support actions in this period. Also excluded were the companions of women with multiple gestations and the companions of women who died or of women whose fetus or newborn died. 

Considering that the maternity hospitals selected for the study allow the presence of a companion during labor, birth or the cesarean section, the sample was calculated based on the number of births in 2013 for each one of the Maternity Hospitals (Maternity Hospital A - 3508; Maternity Hospital B - 3759; Maternity Hospital C - 1525). For the sample calculation for each maternity hospital, presumed prevalence was estimated at 50%, a confidence interval of 95% and a maximum error of 5%, resulting in 346 interviewees in Maternity Hospital A; 349 companions in Maternity Hospital B; and 307 in Maternity Hospital C. As a result, the estimated sample for the study was 1002 companions; in the event, however, interviews were held with 1147 companions due to the availability of funding for undertaking the project. 

Data collection was undertaken in March 2015 - May 2016, using a questionnaire made up of identification variables; sociodemographic characteristics; information on the experience; and support actions for the woman during labor, birth, cesarean section and in the postpartum period. The questionnaire was revised, and - after the end of the testing stage - software was developed to facilitate the recording of the data. The computerized system consisted of a platform in which the data were stored digitally. The interviews were saved in the format of csv files, used by the Microsoft Office Excel Program®. 

After theoretical and practical training, each interviewer received a netbook with the software installed. The interviewers were placed and supervised in the maternity hospitals by the main researcher. The interviews took place in the maternity ward in each maternity hospital, when it was convenient for the companion. The majority of interviews took place outside the birthing room, away from the woman’s room on the ward, without the puerperal woman influencing the answers. 

Each interviewer stored the files on a memory stick and updated the data migration system online so that the information would be sent to the central database. To ensure the quality of the information obtained, and minimize random or systematic errors during data collection, certain procedures were adopted: the use of a checklist with inclusion and exclusion criteria for selecting research subjects; monitoring of data collection throughout the fieldwork until the sample for each institution had been completed; and daily online assessment of the quality of the recording of the data. In addition to this, at the end of data collection, some questions from the questionnaire were repeated via telephone contact in a sample of 5% of the companions interviewed in each maternity hospital. 

The variables analyzed in the present study are: sex (male, female), age (≤ 19, 20 - 59, ≥ 60), skin color (white, black, mixed black-white, others), educational level (none, primary/junior high incomplete, primary/junior high complete, senior high complete, higher education complete), occupation (paid work, unpaid work, unemployed, retired), and link with the woman (partner/father of the baby, mother, woman from the social network/family, others). Previous and present participation in the prenatal care, in the triage, in the labor, and the birth, in the cesarean section, and in the postpartum period (yes, no). Participation in courses and/or seminars (yes, no); and knowledge about the Companion’s Law (yes, no). Actions of emotional support during the labor, birth, cesarean section and postpartum period: remained by the woman’s side, encouraged her, calmed her, praised her, caressed her, held her hand (yes, no). Actions of physical support in the labor: walking, changing position, use of the birthing room’s rocking stool, use of the ball, birthing pool, massage (yes, no). Actions of physical support in the birth: helping the woman to position herself (yes, no). Actions of physical support in the postpartum period: movement, eating/drinking, advised the woman to relax, advised regarding breast-feeding or care with the baby, asked about pain or discomfort (yes, no). Actions of informational support in labor, cesarean section and the postpartum period: guidance regarding what was happening (yes, no). Actions of informational support during the birth: guidance about what was happening, advised her to push, advised her regarding breathing (yes, no). Actions of support related to intermediation during labor, birth, cesarean section and the postpartum period: negotiated the woman’s wishes with the health professionals (yes, no). 

For the analysis, the three databases for the maternity hospitals were grouped into a single database and then exported to the SPSS® program, version 20.0, after which the data were analyzed descriptively (absolute and relative frequencies), with the respective confidence intervals (CI 95%). 

The research project was submitted to the Committee for Ethics in Research with Human Beings via *Plataforma Brasil* - a Brazilian unified database for registering studies involving human beings. The project was approved on 24th February 2014, under Certificate of Ethical Appreciation N. 25589614.3.0000.0121. All the study participants signed the Terms of Free and Informed Consent. 

## Results

Of the 1147 interviewees, the majority were male (77.0%), were of adult age (93.9%), stated that they were Caucasian (53.8%), and were undertaking paid work (86.2%). Regarding educational level, the most frequent was Senior High School complete (36.8%). Regarding the link with the woman being accompanied, the majority were the partner/father of the baby (76.7%). In Maternity Hospital A, one finds the highest frequency of adolescents (6.4%), as well as companions whose skin color was self-reported as mixed-race (45.9%). In Maternity Hospital C, the prevalence of the partner/father of the baby as the companion was higher (82.7%), and the companions had higher educational levels (Table 1). 

**Table 1:** Sociodemographic characteristics of the companions in the public maternity hospitals. Florianópolis, SC, Brazil (2015 - 2016)

	Maternity A (n = 357)		Maternity B (n = 421)		Maternity (= 369)		Total (N=1147)
	n	% CI*(95%)	n	% CI*(95%)	n	% CI*(95%)	N (%)
Sex							
Male	263	73.7 (69.1-78.2)	315	74.8 (70.7-79.0)	305	82.7 (78.8-86.5)	883 (77,0)
Female	94	26.3 (21.7-30.9)	106	25.2 (21.0-29.3)	64	17.3 (13.5-21.2)	264 (23,0)
Age							
≤ 19	23	6.4 (3.9-9.0)	23	5.5 (3.3-7.6)	4	1.1 (0.2-2.1)	50 (4,4)
20 - 59	327	91.6 (88.7-94.5)	391	92.9 (90.4-95.3)	359	97.3 (95.6-98.9)	1077 (93,9)
≥ 60	7	2.0 (0.5-3.4)	7	1.7 (0.4-2.9)	6	1.6 (0.3-2.9)	20 (1,7)
Skin color							
White	172	48.2 (43.0-53.4)	230	54.6 (49.9-59.4)	215	58.3 (53.2-63.3)	617 (53,8)
Black	16	4.5 (2.3-6.6)	50	11.9 (8.8-15.0)	33	8.9 (6.0-11.9)	99 (8,6)
Mixed race	164	45.9 (40.8-51.1)	136	32.3 (27.8-36.8)	118	32.0 (27.2-36.7)	418 (36,4)
Oriental or indigenous	5	1.4 (0.2-2.6)	5	1.2 (0.1-2.2)	3	0.8 (0.0-1.7)	13 (1,1)
Educational level							
None	4	1.1 (0.0-2.2)	7	1.7 (0.4-2.8)	6	1.6 (0.3-2.9)	17 (1,5)
Primary/Junior High incomplete	117	32.8 (27.9-37.6)	119	28.3 (23.9-32.6)	65	17.6 (13.7-21.5)	301 (26,2)
Primary/Junior High complete	95	26.6 (22.0-31.2)	116	27.6 (23.3-31.8)	89	24.1 (19.7-28.5)	300 (26,2)
Senior High School complete	117	32.8 (27.9-37.6)	150	35.6 (31.0-40.2)	155	42.0 (37.0-47.0)	422 (36,8)
Higher Education complete	24	6.7 (4.1-9.3)	29	6.9 (4.5-9.3)	54	14.6 (11.0-18.2)	107 (9,3)
Occupation							
Paid work	299	83.7 (79.9-87.6)	363	86.2 (82.9-89.5)	327	88.6 (85.4-91.9)	989 (86,2)
Unpaid work	29	8.1 (5.3-11.0)	40	9.5 (6.7-12.3)	21	5.7 (3.3-8.1)	90 (7,8)
Unemployed	19	5.3 (3.0-7.6)	10	2.4 (0.9-3.8)	16	4.3 (2.2-6.4)	45 (3,9)
Retired	10	2.8 (1.1-4.5)	8	1.9 (0.5-3.2)	5	1.4 (0.2-2.5)	23 (2,0)
Link with the woman							
Partner/father of the baby	262	73.4 (68.8-78.0)	313	74.3 (70.2-78.5)	305	82.7 (78.8-86.5)	880 (76,7)
Mother	51	14.3 (10.6-17.9)	53	12.6 (9.4-15.8)	42	11.4 (8.1-14.6)	146 (12,7)
Woman from the family/social network	43	12.0 (8.7-15.4)	53	12.6 (9.4-15.8)	22	6.0 (3.5-8.4)	118 (10,3)
Others (father, friend, son/daughter)	1	0.3 (0.0-0.8)	2	0.5 (0.0-1.0)	0	0	3 (0,3)

* CI: Confidence Interval

The percentage of companions with previous participation in the prenatal care, in the obstetric triage, in the labor and in the postpartum period was below 30.0%, and in the birth, was only 19.3%. However, current participation in the prenatal care (61.3%) and in the triage (89.9%) rose considerably. Only 8.6% reported having participated in a course or seminar during the pregnancy, and 23.6% were aware of the Companion’s Law. In Maternity Hospital C, the proportion of interviewees with previous experience as a companion was greater (Table 2). 

**Table 2:** Participation in the prenatal care, triage, labor, birth, cesarean section and postpartum period. Florianópolis, SC, Brazil (2015-2016)

Participation	Maternity A (n = 357)		Maternity B (n = 421)		Maternity C (n = 369)		Total N=1147
	n	% (CI*95%)	n	% (CI*95%)	N	% (CI*95%)	N (%)
Previous							
In prenatal care	69	19.3 (15.2-23.4)	100	23.8 (19.7-27.8)	116	31.4 (26.7-36.2)	285 (24.8)
In triage	80	22.4 (18.1-26.7)	89	21.1 (17.2-25.0)	109	29.5 (24.9-34.2)	278 (24.2)
In labor	83	23.2 (18.9-27.6)	82	19.5 (15.7-23.3)	106	28.7 (24.1-33.3)	271 (23.6)
In the birth	67	18.8 (14.7-22.8)	74	17.6 (13.9-21.2)	80	21.7 (17.5-25.9)	221 (19.3)
In the cesarean section	49	13.7 (10.1-17.3)	35	8.3 (5.7-11.0)	40	10.8 (7.7-14.0)	124 (10.8)
In the postpartum period	88	24.6 (20.2-29.1)	102	24.2 (20.1-28.3)	122	33.1 (28.2-37.9)	312 (27.2)
Current							
In the prenatal care	221	61.9 (56.8-67.0)	248	58.9 (54.2-63.6)	268	72.6 (68.1-77.2)	737 (61.3)
In triage	319	89.4 (86.1-92.6)	360	85.5 (82.1-88.9)	341	92.4 (89.7-95.1)	1020 (89.9)
In the labor	357	100.0	421	100.0	369	100.0	1147 (100.0)
In the birth	272	76.2 (71.8-80.6)	321	76.2 (72.2-80.3)	268	72.6 (68.1-77.2)	861 (75.1)
In the cesarean section	85	23.8 (19.4-28.2)	100	23.8 (19.7-27.8)	101	27.4 (22.8-31.9)	286 (24.9)
In the postpartum period	315	88.2 (84.9-91.6)	289	68.6 (64.2-73.1)	342	92.7 (90.0-95.3)	946 (82.5)
Participation in course or seminar	25	7.0 (4.3-9.7)	22	5.2 (3.1-7.4)	52	14.1 (10.5-17.6)	99 (8.6)
Knowledge of the Companion’s Law	64	17.9 (13.9-21.9)	128	30.4 (26.0-34.8)	79	21.4 (17.2-25.6)	271 (23.6)

* CI: Confidence Interval.

During labor, actions of emotional support were more frequent - such as remaining by the woman’s side and calming her, followed by actions of physical support: helping in changing position and walking. In Maternity Hospital C, some actions of physical support had a higher frequency (Table 3). 

**Table 3:** Actions of support in labor, in public maternity hospitals. Florianópolis, SC, Brazil (2015-2016)

Actions of support	Maternity A (n = 357)		Maternity B (n = 421)		Maternity C (n = 369)		Total N = 1147
	n	%(CI*95%)	n	%(CI*95%	n	%(CI*95%)	N (%)
Emotional							
Remained by the woman’s side	356	99.7 (99.2-100.3)	414	98.3 (97.1-99.6)	367	99.5 (98.7-100.2)	1137 (99.1)
Encouraged her	346	96.9 (95.1-98.7)	413	98.1 (96.8-99.4)	364	98.6 (97.5-99.8)	1123 (97.9)
Calmed her	348	97.5 (95.8-99.1)	413	98.1 (96.8-99.4)	365	98.9 (97.8-100.0)	1126 (98.2)
Praised her	306	85.7 (82.1-89.3)	379	90.0 (87.1-92.9)	339	91.9 (89.1-94.7)	1024 (89.3)
Caressed her	333	93.3 (90.7-95.9)	393	93.3 (91.0-95.7)	358	97.0 (95.3-98.7)	1084 (94.5)
Held hand	341	95.5 (93.4-97.7)	412	97.9 (96.5-99.2)	354	95.9 (93.9-97.9)	1107 (96.5)
Physical							
Walking	285	79.8 (75.7-84.0)	360	85.5 (82.1-88.9)	323	87.5 (84.1-90.9)	968 (84.4)
Changing position	315	88.2 (84.9-91.6)	378	89.8 (86.9-92.7)	344	93.2 (90.6-95.8)	1037 (90.4)
Use of the rocking stool	103	28.8 (24.1-33.6)	39	9.3 (6.5-12.0)	120	32.5 (27.7-37.3)	262 (22.8)
Use of the ball	78	21.8 (17.6-26.1)	190	45.1 (40.4-49.9)	243	65.8 (61.0-70.7)	511 (44.6)
Birthing pool	184	51.5 (46.3-56.7)	333	79.1 (75.2-83.0)	291	78.9 (74.7-83.0)	808 (70.4)
Massage	199	55.7 (50.6-60.9)	274	65.1 (60.5-69.6)	272	73.7 (69.2-78.2)	745 (65.0)
Informational							
Advised the woman on what was happening	264	74.0 (69.4-78.5)	342	81.2 (77.5-85.0)	301	81.6 (77.6-85.5)	907 (79.1)
Intermediation							
Negotiated the woman’s wishes	177	49.6 (44.4-54.8)	233	55.3 (50.6-60.1)	288	78.0 (73.8-82.3)	698 (60.9)

* CI: Confidence Interval.

The support actions undertaken during the birth and the cesarean section are described below. In the birth, the physical support was characterized by assisting the woman in positioning herself, this being undertaken by 65.0% of the companions; the informational support was characterized as providing guidance on what was happening in the birth (74.7%), advising the woman to push (85.4%) and providing guidance on breathing (77.4%). Support in relation to intermediation was undertaken by only 56.7% of the companions. Among the participants who were present during the cesarean section, most undertook actions of emotional support. In the present study, the companions were not questioned about physical support during the cesarean section, as there is no freedom for the woman to undertake any activity whatsoever during the surgical procedure. Among the maternity hospitals studied, Maternity Hospital C presented results with high percentages in relation to support related to intermediation, as more than half of the companions reported negotiating the woman’s wishes with the health professionals, both in labor and during the cesarean section (Table 4). 

**Table 4:** Actions of support in birth and cesarean section in Public Maternity Hospitals. Florianópolis, SC, Brazil (2015-2016)

Actions of support	Maternity A (n = 357)		Maternity B (n = 421)		Maternity C (n = 369)		Total (1147)
	n	% (CI*95%)	n	% (CI*95%)	n	% (CI*95%)	N (%)
Birth (n=861)							
Emotional							
Remained at the woman’s side	256	94.1 (91.3-96.9)	312	97.2 (95.4-99.0)	266	99.2 (98.2-100.3)	834 (98.9)
Encouraged her	258	94.9 (92.2-97.5)	306	95.3 (93.0-97.6)	262	97.8 (96.0-99.5)	826 (95.9)
Calmed her	260	95.6 (93.1-98.0)	307	95.6 (93.4-97.9)	259	96.6 (94.5-98.8)	826 (95.9)
Praised her	219	80.5 (75.8-85.2)	278	86.6 (82.9-90.3)	249	92.9 (89.8-96.0)	746 (86.6)
Caressed her	231	84.9 (80.7-89.2)	286	89.1 (85.7-92.5)	254	94.8 (92.1-97.4)	771 (89.6)
Held her hand	239	87.9 (84.0-91.8)	290	90.3 (87.1-93.6)	247	92.2 (88.9-95.4)	776 (90.1)
Physical							
Helped her to position herself	180	66.2 (60.5-71.8)	191	59.5 (54.1-64.9)	189	70.5 (65.0-76.0)	560 (65.0)
Informational							
Advised her on what was happening	189	69.5(64.0-75.0)	242	75.4 (70.4-80.1)	212	79.1 (74.2-84.0)	643 (74.7)
Told her to push	226	83.1 (78.6-87.6)	276	86.0 (82.2-89.8)	233	86.9 (82.9-91.0)	735 (85.4)
Advised her on breathing	203	74.6 (69.4-79.8)	247	77.0 (72.3-81.6)	216	80.6 (75.8-85.3)	666 (77.4)
Intermediation							
Negotiated the woman’s wishes	131	48.2 (42.2-54.1)	158	49.2 (43.7-54.7)	199	74.2 (69.0-79.5)	488 (56.7)
Cesarean (n = 286)							
Emotional							
Remained at the woman’s side	81	95.3 (90.7-99.8)	98	98.0 (95.2-100.8)	100	99.0 (97.1-100.9)	279 (97.6)
Encouraged her	72	84.7 (77.0-92.4)	93	93.0 (88.0-98.0)	96	95.0 (90.8-99.3)	261 (91.3)
Calmed her	78	91.8 (85.9-97.7)	95	95.0 (90.7-99.3)	98	97.0 (93.7-100.4)	271 (94.8)
Praised her	64	75.3 (66.0-84.6)	80	80.0 (72.1-87.9)	87	86.1 (79.3-92.9)	231 (80.8)
Caressed her	69	81.2 (72.8-89.6)	88	88.0 (81.6-94.4)	90	89.1 (83.0-95.2)	247 (86.4)
Held her hand	58	68.2 (58.2-78.2)	90	90.0 (84.1-95.9)	79	78.2 (70.1-86.3)	227 (79.4)
Informational							
Advised her on what was happening	57	67.1 (57.0-77.1)	71	71.0 (62.0-80.0)	75	74.3 (65.6-82.9)	203 (71.0)
Intermediation							
Negotiated the woman’s wishes	29	34.1 (23.9-44.3)	35	35.0 (25.6-44.4)	57	56.4 (46.7-66.2)	121 (42.3)

* CI: Confidence Interval.

In the postpartum period, the emotional dimension was also that which received the greatest emphasis, with frequencies above 90% in all actions. Helping in the care with the baby (94.8%) and advising the woman to relax (93.2%) were the actions of physical support undertaken most by the companions. More than half of them negotiated the woman’s wishes with the health professionals (64.4%). In Maternity Hospital C, the prevalences of informational support and support related to intermediation were higher than in Maternity Hospitals A and B (Table 5). 

**Table 5:** Actions of support in the postpartum period in public maternity hospitals. Florianópolis, SC, Brazil (2015-2016)

Actions of Support	Maternity A (n = 315)		Maternity B (n = 289)		Maternity C (n = 342)		Total N= 946
	n	%(CI*95%)	n	%(CI*95%)	n	%(CI*95%)	N (%)
Emotional							
Remained at the woman’s side	313	99.4 (98.5-100.2)	282	97.6 (95.8-99.4)	340	99.4 (98.6-100.2)	935 (98.8)
Encouraged her	285	90.5 (87.2-93.7)	277	95.8 (93.5-98.1)	330	96.5 (94.5-98.4)	892 (94.3)
Calmed her	298	94.6 (92.1-97.1)	279	96.5 (94.4-98.6)	336	98.2 (96.8-99.6)	913 (96.5)
Praised her	268	85.1 (81.1-89.0)	275	95.2 (92.7-97.6)	317	92.7 (89.9-95.5)	860 (90.9)
Caressed her	281	89.2 (85.8-92.6)	264	91.4 (88.1-94.6)	330	96.5 (94.5-98.4)	875 (92.5)
Physical							
Movement	260	82.5 (78.3-86.7)	237	82.0 (77.6-86.4)	303	88.6 (85.2-92.0)	800 (84.6)
Eating/drinking	244	77.5 (72.8-82.1)	242	83.7 (79.5-88.0)	267	78.1 (73.7-82.5)	753 (79.6)
Advised to relax	290	92.1 (89.1-95.1)	265	91.7 (88.5-94.9)	327	95.6 (93.4-97.8)	882 (93.2)
Helped in breast-feeding	250	79.4 (74.9-83.8)	245	84.8 (80.6-88.9)	292	85.4 (81.6-89.1)	787 (83.2)
Helped in the care with the baby	287	91.1 (88.0-94.3)	282	97.6 (95.8-99.4)	328	95.9 (93.8-98.0)	897 (94.8)
Asked about pain or discomfort	262	83.2 (79.0-87.3)	264	91.3 (88.1-94.6)	320	93.6 (91.0-96.2)	846 (89.4)
Informational							
Provided advice on what was happening	195	61.9 (56.5-67.3)	214	74.0 (69.0-79.1)	277	81.0 (76.8-85.2)	686 (72.5)
Intermediation							
Negotiated the woman’s wishes	164	52.1 (46.5-57.6)	168	58.1 (52.4-63.8)	277	81.0 (76.8-85.2)	609 (64.4)

* CI: Confidence Interval

## Discussion

The results show that, although the majority of companions had no previous experience of supporting the woman during labor, birth, cesarean section and the postpartum period, and had practically no preparation during the prenatal care, they took on the role of provider of support in the four dimensions analyzed (emotional, physical, informational and related to intermediation).

The participation of the partner/father of the baby in the role of companion was similar to that found in other studies with quantitative^8^ ^,^ ^14^ ^-^ ^15^ and qualitative^6^ ^,^ ^10^ ^,^ ^16^ approaches. The presence of the father in this scenario symbolizes - even if only partially - the family’s becoming closer after the birth. International studies have revealed that in other countries, the presence of the father during the birth is accepted^17^, regardless of whether this is related or not to the provision of support, it frequently being the case that the doula or midwife takes on this task^18^ ^-^ ^19^.

Another relevant aspect is knowledge of the Companion’s Law, as this information can contribute to the woman and her companion demanding their rights from the very first moment of obstetric inpatient treatment. Although this document^6^ was published in 2005, its limited publicizing has stopped it from being used as an instrument for ensuring the presence of the companion^6^ ^-^ ^7^. 

The fact that few companions participated in courses and/or seminars during the pregnancy, as well as not having previous experience, may have influenced their ignorance of their rights. However, these aspects did not impede or restrict the companion from performing his or her role as provider of support to the woman, especially in relation to the emotional dimension. Providing emotional support was also mentioned in other studies as activities which calm, encourage, transmit security and mitigate the woman’s pain^6^ ^,^ ^10^ ^,^ ^17^ ^,^ ^20^ ^-^ ^22^. 

In relation to the actions of physical support in the labor, the activities of assistance in changing position, in walking and in using the birthing pool were mentioned by the majority of companions - that is, a large proportion of the interviewees helped in the woman’s free movement. This practice must be encouraged during labor, as it allows the woman to adopt the position that she finds the most comfortable, should there be no clinical contraindication^23^.

The actions of support undertaken by the companion are considered to be nonpharmacological methods of relieving pain and anxiety - and can, therefore, reduce the duration of labor^23^ ^-^ ^25^. As a result, it may be inferred that the companion is contributing to implementing good practices in childbirth care, as he or she encourages and helps the parturient woman to undertake the recommended activities. 

During the cesarean section, the reduction in the frequency of actions of support undertaken was notable - principally those related to information and intermediation. This finding may result from the fear and apprehension resulting from the need for the surgical procedure^22^ - or from the restriction on actions that the environment itself imposes upon the layperson. As a result, the companion takes on a more passive role, due to the lack of preparation and advice, in addition to the insecurity he or she feels in relation to providing support. In some maternity hospitals, the companion is prevented from participating in the birth during the surgical procedure due to being prohibited from doing so by the health professionals^22^. The members of the health team reinforce that this is no place for the companion, justifying the statement by indicating that he is not familiar with the medical routines and does not know how to behave^26^.

The frequent participation of the companions in the postpartum period is similar to that found in another study undertaken in Santa Catarina, Brazil^27^; however, this is not the reality found in other Brazilian maternity hospitals^8^. As a result, these women are deprived of the support of a person from their social network, who could assist in care with the baby and in movement. Few works have focused on this period: actions of emotional support, such as caressing, staying by the woman’s side and calming her are mentioned most^6^ ^,^ ^13^. In the dimension of physical support, the puerperal women and the companions mainly report the importance of assisting in care with the baby and in breast-feeding^6^ ^,^ ^13^ ^,^ ^15^. 

It is emphasized that, through participating actively in the breast-feeding process, the companion is supporting and encouraging the woman, causing her to feel more confident for establishing this process. Participating in the care with the baby in the Maternity Hospital is consistent with the current paradigm, which places the baby’s father as a fundamental element for humanized birth, and promotes the man’s greater involvement as a caregiver. 

The actions of informational support were identified in all the periods evaluated, mainly during labor and birth. The companion may keep the woman informed during these periods regarding breathing, when to push for the baby’s expulsion, regarding the progress of the labor, and what is happening in the birth^6^ ^,^ ^13^ ^,^ ^28^. In addition to this, he can reinforce the information from the health professionals relating to the procedures being undertaken^13^. These actions of the companion’s contribute to the woman feeling encouraged and, consequently, having a calmer and more pleasurable experience. 

Support in terms of intermediation was mentioned least by the companions, showing the difficulties they have in negotiating the woman’s wishes with the health professionals. This may be associated with the women’s and the companions’ fear of suffering some sort of repression from the health professionals if they were to express wishes which could interfere in the hospital routines. This is although the presence of the companion is indicated as a practice which contributes to reducing institutional violence, as he can act as a defender of the woman and protect her against maltreatment^9^ ^,^ ^29^.

 The Born in Brazil Survey indicated that women who went into labor in public services had a higher probability of suffering physical, verbal or psychological violence at the time of birth, in comparison with those who did not go into labor, or who did so in the supplementary services’ maternity hospitals. However, one protective factor for mitigating this risk, regardless of having experienced labor or not, or of the modality of the service, is the presence of the companion^29^. This being the case, it is clear that embracing the woman and the companion makes open dialogue with the health team permissible, agreeing with the principle of the woman’s autonomy and the humanization of birth.

One of the points emphasized in this work is the difference between the prevalences of the actions of physical support and of intermediation in the three separate maternity hospitals, although all are part of the SUS. This analysis confirms the need to assess the historical, political and structural context of each maternity hospital in order to understand which focuses are valued in the obstetric care - such an evaluation was not the object of the present study. Nevertheless, it is important to highlight that the actions of emotional and informational support had highly similar frequencies in the three maternity hospitals; this aspect represents positive significance.

Intermediation support had higher prevalence in Maternity Hospital C, which presents relevant characteristics for the practice of humanization of birth, such as the Galba de Araújo Prize and the right to the presence of a companion since this was made law^13^. It is advisable that the dialogue between the health professionals and the companion should take place starting during the pregnancy, informing him or her regarding the characteristics of the maternity hospital in which he or she will accompany the birth. It is possible, in this way, to investigate what the expectations of the woman and the companion regarding the birth are, and what negotiations could be undertaken to meet the needs of the parturient woman^30^.

The panorama presented situates the companion as an important provider of support to the woman during the periods of the obstetric inpatient treatment, mainly in the emotional dimension, promoting times of well-being when he or she stays by her side, calms her and encourages her. Regarding physical comfort, the encouragement to use noninvasive technologies during labor was evidenced, as stipulated in the principles of humanization, and in the strategies for nonpharmacological pain relief. The actions of informational support were shown to be more accentuated at the time of birth, which may be related to the health professionals’ guidance to the woman to assist in the birth and, thus, were reinforced by the companions. In comparison with the other dimensions of support, negotiating the woman’s wishes with the health professionals, in all periods evaluated, showed a low frequency - this observation may be linked to the sometimes less-than-welcoming relationship which is established between the health professionals and the companion.

The following are considered to be limitations of the study. The content of the courses and seminars attended by the companions was not assessed; neither were the wishes of the woman, which the companion negotiated with the health professionals. In spite of this, the study is innovative and presents data regarding the actions of support undertaken by the companion in the emotional, physical, emotional and intermediation-related dimensions, which had not yet been broadly assessed. 

## Conclusion

The quantitative analysis of the actions of support in labor, birth, cesarean section and the postpartum period made it possible to identify the companion as an active partner throughout the process, and not merely a spectator. 

The actions of support in the emotional dimension presented higher percentages, demonstrating that the companion feels more comfortable and better able to provide this form of support. In the physical dimension, during labor and birth, emphasis is placed on changing position and assistance for the birth position. In the postpartum period, all the actions of physical support were undertaken by most of the interviewees - in particular, care with the baby. The actions of informational support were undertaken more frequently during labor and birth. Support related to intermediation had the lowest percentage during cesarean section. 

Considering the Brazilian context, this study’s findings contribute to strengthening the importance of the participation of the companion from the woman’s social network, given that this provider of support does not entail costs for the parturient woman. As a result, it is necessary to advise the companion in the prenatal period regarding the progression of labor and birth, in order to add to the actions of support in the informational dimension. 

Support related to intermediation is closely associated with communication between the woman and the companion, and - later - with negotiation between the companion and the health professionals. This interaction can be harmonious, to the extent that the health professionals perceive the importance of promoting the woman’s autonomy during labor and birth, integrating the companion into the identification and requesting of her needs. 

The actions of support undertaken in the postpartum period show that the presence of the companion is of great importance, as he or she contributes directly to the woman’s comfort. In taking on the function of support provided in this period, the companion becomes closer to the woman and the newborn, facilitating the parental transition - in particular when the companion is the baby’s father.
